# Structural and functional brain abnormalities in children with schizotypal disorder: a pilot study

**DOI:** 10.1038/s41537-020-0095-7

**Published:** 2020-03-18

**Authors:** Ya Wang, Ian H. Harding, Renee Testa, Bruce Tonge, Harvey Jones, Marc Seal, Nola Ross, Raymond C. K. Chan, Florian van Beurden, Ahmad Abu-Akel, Efstratios Skafidas, Christos Pantelis

**Affiliations:** 1 Melbourne Neuropsychiatry Centre, Department of Psychiatry, The University of Melbourne & Melbourne Health, Carlton South, VIC Australia; 2 Neuropsychology and Applied Cognitive Neuroscience Laboratory, CAS Key Laboratory of Mental Health, Institute of Psychology, Beijing, China; 3 Turner Institute for Brain and Mental Health & School of Psychological Sciences, Monash University, Clayton, VIC Australia; 4 School of Psychological Sciences, Monash University, Clayton, VIC Australia; 5 The Child and Adolescent Neuropsychology Group, East Melbourne, VIC Australia; 6 Mental Health Department, The Royal Children’s Hospital, Parkville, VIC Australia; 7 Centre for Developmental Psychiatry and Psychology, School of Clinical Sciences, Monash University, Clayton, VIC Australia; 8 Department of Psychology, Epworth HealthCare, Richmond, VIC Australia; 9 Murdoch Children’s Research Institute, Parkville, VIC Australia; 10 Institute of Psychology, University of Lausanne, Lausanne, Switzerland; 11 Department of Electrical and Electronic Engineering, The University of Melbourne, Parkville, VIC Australia; 12 Florey Institute for Neurosciences and Mental Health, Parkville, VIC Australia

**Keywords:** Schizophrenia, Psychosis, Biomarkers

## Abstract

Schizotypal disorder lies in the schizophrenia spectrum and is widely studied in adult populations. Schizotypal disorder in children (SDc) is less well described. This study examined brain morphological and functional connectivity abnormalities in SDc (12 SDc and 9 typically developing children), focusing on the default mode and executive control brain networks. Results indicated that SDc is associated with reduced grey matter volume (GMV) in superior and medial frontal gyri, and increased resting-state functional connectivity between the superior frontal gyrus and inferior parietal lobule, compared to typically developing children (cluster-level FWE-corrected *p* < 0.05). The brain structure abnormality (GMV in left superior frontal gyrus) was correlated with clinical symptoms in SDc (*r* = −0.66, *p* = 0.026) and functional connectivity abnormality was correlated with extra-dimensional shifting impairments in all participants (*r* = 0.62, *p* = 0.011), suggesting their contribution to the underlying mechanisms of clinical presentation. These preliminary results motivate further work to characterize the neural basis of SDc and its significance as a risk factor for later psychosis.

## Introduction

Schizotypal disorder (SD) lies within the schizophrenia spectrum and represents one of the high-risk groups for schizophrenia.^1–3^ There is a growing interest in childhood manifestations of schizotypal disorder (SDc),^4,5^ which are characterized by bizarre magical and paranoid fantasies and perceptual disturbances preoccupying the internal world of the child.^2–4^ These preoccupying thoughts interfere with normal social interaction and activities, and cause anxiety and distress.^5^ SD has been identified in children as early as 5–6 years old.^4,6^ The prevalence of SDc is not well established. Population studies suggest a prevalence of 5.9% with “at least one definite” psychotic symptom in a UK cohort of 2127 twin children aged 12 years^7^ and 10.2% with ”strong symptoms” of psychosis in a population of 27,000 Australian children (mean age 11.91 years).^8^ Two small longitudinal studies indicated a 17–25% risk for youth with SD developing a psychosis within 3 years.^6,9^ Detailed study of SDc is likely to contribute to further recognition and definition of this pathology, while contributing to further understanding of the schizophrenia spectrum and the prediction of frank onset of psychosis.

The preoccupation with internal fantasies in SDc is thought to reflect a difficulty in switching attention from internal to external foci.^5^ This proposition motivates corresponding neurobiological hypotheses of dysfunction in relevant brain networks. Studies in healthy participants have consistently shown that when an individual is engaged in internally-oriented processes, the default mode network (DMN, including medial prefrontal cortex, posterior cingulate cortex, medial temporal lobe, and angular gyrus) is activated and the executive control network (ECN, including dorsal lateral prefrontal cortex, and superior parietal lobules) is deactivated;^10,11^ the opposite pattern is evident during externally-oriented and goal-directed tasks.^12^ The DMN and ECN are affected in schizophrenia patients and adults with SD, both structurally and functionally.^13,14^ Adults with SD are consistently reported to have reduced grey matter volume (GMV) in the medial temporal lobe, with more variable evidence of prefrontal cortical involvement.^15^ Functionally, abnormalities within the DMN have also been reported in individuals with SD, including both increased and decreased resting-state functional connectivity compared to controls.^16^ During cognitive tasks, individuals with SD also showed different profiles of activation within the ECN compared to controls.^17–19^ No study has yet been conducted to examine the neural basis of SDc. This study hypothesized that brain structural (GMV) abnormalities would manifest in SDc, relative to typically developing children, within the DMN and ECN, and that these structural changes would be associated with large-scale functional (resting-state connectivity) impairments.

## Results

SDc had higher Melbourne Assessment of Schizotypy in Kids (MASK) scores, completed fewer intra-/extra-dimensional set-shifting task (IDED) stages, and committed more IDED extra-dimensional errors than typically-developing children (TDC) (see Supplementary Table 1).

**Fig. 1:**
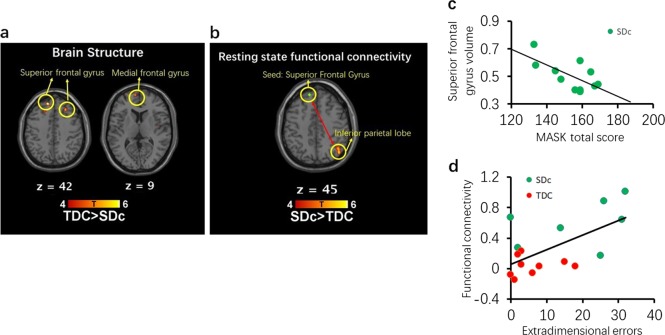
Brain imaging and correlation results. **a** Structural difference between SDc and TDC. **b** Resting-state functional connectivity difference between SDc and TDC. Seed region: superior frontal gyrus (centered at [−12 45 42]). **c** The relationship between MASK total score and left superior frontal gyrus (centered at [−12 45 42]) volume in SDc individuals. **d** The relationship between extra-dimensional errors and functional connectivity between superior frontal gyrus (centered at [−12 45 42]) and inferior parietal lobule in all participants.

SDc showed significantly reduced GMV within the DMN, including the superior frontal gyrus (BA8: peak MNI coordinates: [−12,45,42], *t* = 7.06, *p*_corrected_ = 0.042) and [27,32,42], *t* = 5.40, *p*_corrected_ = 0.043) and the medial frontal gyrus (BA10: [−8,62,9], *t* = 5.32, *p*_corrected_ = 0.050) compared to TDC (Fig. 1a and Supplementary Table 2). Uncorrected effect-size maps (Cohen’s *d* > 0.8, representing “large” effects) are additionally presented in Supplementary Fig. 1, pointing to potentially more extensive DMN and ECN involvement beyond the power of this pilot work to infer definitively.

SDc also exhibited statistically significantly increased functional connectivity between the superior frontal gyrus (seed [−12,45,42]) and the inferior parietal lobule (BA39: [45, −66, 45]) (a region within ECN) relative to TDC, *t* = 6.01, *p*_corrected_ < 0.001 (see Fig. 1b and Supplementary Table 2). Effect-size maps (Cohen’s *d* *>* 0.8) provided preliminary indications of widespread abnormalities in DMN-ECN interactions (Supplementary Fig. 2).

The GMV of the left superior frontal gyrus, centered at [−12,45,42], was inversely correlated with the MASK total score (*r* = −0.66, *p* = 0.026) in SDc (see Fig. 1c). Functional connectivity between the superior frontal gyrus and the inferior parietal lobule was significantly correlated with IDED stages completed (Spearman correlation, *r*_s_ = −0.56, *p* = 0.023) and extra-dimensional errors (*r* = 0.62, *p* = 0.011) (See Fig. 1d).

## Discussion

In this study, we examined the neural impairments in SDc. We identified brain structural and functional abnormalities of SDc and suggest these abnormalities may be related to the cognitive and clinical symptoms of SDc.

SDc exhibited reduced GMV in the superior and medial frontal gyrus, and the GMV in the superior frontal gyrus was correlated with clinical symptoms in SDc. These results suggested the importance of frontal dysfunction in SDc, particularly in regions of the DMN. The decreased GMV in frontal regions are consistent with those findings reported in patients^14,20^ and high-risk individuals for schizophrenia.^21,22^ However, these results diverge from previous reports of SD in adults, where prefrontal cortex volumes were relatively preserved or even increased.^1^ This distinction may reflect the developmental status of our younger cohort, particularly given the known non-linear trajectories of brain maturation and decline in neurodevelopmental diseases.^23,24^ Alternatively, or consequentially, SD may manifest differently in childhood and adulthood. Critically, the observed correlation between GMV and MASK total score is consistent with similar relationships between clinical symptoms and GMV in prefrontal regions in high-risk individuals for psychosis.^25^

Increased functional connectivity between the superior frontal gyrus and inferior parietal lobule was also observed in SDc, and increased functional connectivity was correlated with poorer attentional set-shifting performance in all participants. These results suggest that the increased functional connectivity in SDc is maladaptive and related to their reduced cognitive control. This abnormal functional connectivity is also consistent with previous studies examining the relationship between DMN and ECN in schizophrenia and at-risk individuals.^26–28^ The DMN is related to processing internal and self-related information,^10^ while the ECN is implicated in external information processing and goal-directed regulation.^12^ While further study is required, the abnormal functional interactions observed between these systems provides a potential mechanism underpinning preoccupation with internal fantasies in SDc. One possibility is that the inability to effectively de-couple the DMN and ECN would make it difficult for SDc to differentiate the perception of external stimuli from representations of internal information,^13^ causing deficits in monitoring reality, abnormal self-awareness, and altered self-experience.^13,27^ The correlation between DMN-ECN functional dysconnectivity and attentional set-shifting abilities further supports the link between this biological abnormality and impaired switching between internally-focused and externally-focused attention.

There are several limitations in this study: First, given it is a preliminary study, the sample size is small. Larger samples will be necessary to corroborate and extend these findings. Second, this is a cross-sectional study and only include children with SD. Further longitudinal studies and studies recruiting both children and adults with SD may illustrate the clinical and biological trajectories of SD, particularly with respect to risk for psychosis. Third, this study only examined brain structure and resting-state functional connectivity. Task-based functional imaging studies that explicitly manipulate internal and external attention processes will also be important for further defining the neurofunctional substrates of SDc expression.

Taken together, this study represents an initial step in examining the neural basis of schizotypal disorder in childhood and motivates further work towards its characterization within the schizophrenia spectrum.

## Methods

### Participants

Twelve SDc (7 males) and 9 TDC (6 males) were recruited from two neurodevelopmental disorders clinics in Melbourne and from the general community. The mean age of SDc and TDC were 12.15 (SD = 2.67) and 13.49 (SD = 1.85) years respectively. SDc (DSM-IV-TR criteria) was diagnosed using the MASK a validated scale based on interviews with both the children and their parents.^4^ A diagnostic cutoff of 132 was used, based on sensitivity/specificity determined during psychometric development of the MASK.^4^ Demographic and clinical information are provided in Supplementary Table 1. This study was approved by Melbourne Health Research Ethics Committee and Monash University Research Ethics Committee. Written informed consent was provided by the guardians of the participants.

### Measures

The MASK is a standardized clinical assessment for SDc^4^ and includes a screening checklist of 57 items comprising two factors: social/pragmatic symptoms, and positive schizotypal symptoms.^4^ It has been shown to be valid in distinguishing SDc from both autism spectrum disorder and TDC.^4^

Set-shifting and reversal learning impairments are among the most pervasive cognitive symptoms in patients with schizophrenia and adult patients with SD,^29,30^ and are directly related to ECN function.^5,31,32^ The IDED from the Cambridge Neuropsychological Test Automated Battery (CANTAB) was therefore used to measure set-shifting and reversal learning ability.^33^ The number of stages completed, intra-dimensional errors, extra-dimensional errors, and extra-dimensional reversal errors were recorded.

### Imaging acquisition

Neuroimaging data were acquired on a 3T Siemens Trio Scanner (Siemens, Erlangen, Germany) with a 32-channel RF head coil at the Royal Children’s Hospital, Melbourne. High resolution anatomical data were acquired via a T1-weighted MPRAGE sequence using the following parameters: 176 sagittal brain slices; TR = 2530 ms; TE = 1.74 ms; FOV = 256 mm; matrix = 256 × 256; flip angle = 7°, voxel size = 1 × 1 × 1 mm. Blood oxygen-level dependent (BOLD) functional data were acquired using gradient recalled-echo echo planar images (GRE-EPI) while the participant was at rest with eyes open, using the following parameters: 60 axial brain slices, multi-band acceleration factor 3, TR = 1500 ms, TE = 33 ms, flip angle = 85°, FOV = 255 mm, matrix = 255*255, voxel size = 2.5 × 2.5 × 2.5 mm. A total of 180 whole-brain volumes were collected. The scanning time (including T1 and resting-state fMRI) was about 12 min.

### Image processing

The structural images were analyzed using voxel-based morphology (VBM) with diffeomorphic anatomical registration through exponentiated lie algebra (DARTEL) normalization as embedded in SPM12 (http://www.fil.ion.ucl.ac.uk/spm/). The T1 images were segmented, normalized, modulated to preserve volume encoding, and smoothed with a full width at half maximum (FWHM) kernel of 5 mm. The group comparison of GMV was conducted in SPM with *t*-test while controlling for confounding due to sex, age, and whole brain volume variables.

Resting-state fMRI data were preprocessed using the DPABI toolbox^34^ for SPM12. Preprocessing included slice timing correction, rigid-body realignment, coregistration of functional to structural data, normalization to standard (MNI) space, spatial smoothing using a FWHM 4 mm kernel, and temporal filtering between 0.01 and 0.1 Hz. Regression was used to estimate and account for variance attributable to head motion (Friston’s 24 parameters), white matter and CSF signal. Seed-based functional connectivity was undertaken on the residuals. The seeds were defined by spherical ROIs (4 mm radius) centered on brain regions showing a between-group GMV difference.

Inference of between-group differences in GMV and functional connectivity were restricted to a mask of the DMN and ECN, defined based on a published network parcellation,^35^ for both structure and resting-state data analyses. Multiple comparison correction within this mask was performed using a cluster-level FWE correction at *p* < 0.05 threshold in both cases.

The GMV and functional connectivity estimates in brain areas which showed group differences were extracted (with 4 mm radius sphere ROI) and correlated with (Pearson correlation if data was normally distributed and Spearman correlation if data were not normally distributed) MASK total score in SDc and IDED measures (stages completed, ID error, ED error, extra-dimensional reversal error) in all participants. A standard significance threshold of *p* < 0.05 was adopted. For more details of image processing, see Supplementary Method.

### Reporting summary

Further information on research design is available in the Nature Research Reporting Summary linked to this article.

## Supplementary information

Supplementary_materials

Reporting Summary

## Data Availability

The data that support the findings of this study are available from the corresponding author upon reasonable request.

## Code availability

We used standardized toolbox to analyze the data, no special code was used in data analysis.
