# Supplementary material for: Structural and functional brain abnormalities in children with schizotypal disorder: a pilot study
Source: NPJ Schizophr. 2020 Mar 18;6:6. doi: 10.1038/s41537-020-0095-7 (PMC7080771; doi:10.1038/s41537-020-0095-7)

Structural and functional brain abnormalities in children with schizotypal disorder: A pilot study

Ya Wang[1,2], Ian H. Harding[1,3], Renee Testa[1,4,5,6], Bruce Tonge[7], Harvey Jones[8], Marc Seal[9], Nola Ross[5], Raymond C.K. Chan[2], Florian van Beurden[1], Ahmad Abu-Akel[10], Efstratios Skafidas[1,11], Christos Pantelis[1,11,12, *]

Supplementary method

Detailed description of image processing

The structural images were analyzed using voxel-based morphology (VBM) with diffeomorphic anatomical registration through exponentiated lie algebra (DARTEL) normalization as embedded in SPM12 (http://www.fil.ion.ucl.ac.uk/spm/) running on MATLAB2017b (Mathworks Inc., Sherborn, MA). The T1 image of each participant was first segmented into grey matter, white matter and cerebrospinal fluid, then all participants' images were used to create and warp to a study-specific template before normalization to MNI space. The normalized grey matter partial volume images were modulated by the Jacobian of the combined warp from native to study to MNI space, such that the intensity of each voxel in the final images encoded grey matter volume. The images were then smoothed with full width at half maximum (FWHM) kernel of 5 mm. The group comparison (t-test) of grey matter volume was conducted in SPM while controlling for sex, age and total

brain volume. Inference was restricted to a mask of the DMN and ECN, defined based on Shirer et al.'s [1] network parcellation. Multiple comparison correction within this mask was performed using a cluster-level FWE correction at $p<0.05$ threshold.

Resting state fMRI data were preprocessed using the DPABI toolbox [2] for SPM12. The first 5 volumes were removed for signal equilibrium. Preprocessing included slice timing correction, rigid-body motion realignment, coregistration of the functional EPIs to the structural T1 data, segmentation and normalization of the T1 and normalization of the EPI images to MNI space; spatial smoothing using a FWHM 4 mm kernel; and temporal filtering between 0.01 and 0.1 Hz. In this study, all but two individuals showed head motion of no greater than 2 mm or 2 degrees. The remaining two moved, respectively, 3.3 mm/3.0 degrees and 3.1 mm/3.9 degrees. To robustly account for and mitigate the influence of variance in the data introduced by head motion, we employed a conservative data cleaning approach: 24 parameters were regressed from the data in each individual, corresponding to the 6 head motion parameters (translation in 3 planes, rotation in 3 axes) at each time point, the 6 parameters of the previous timepoint, plus each of these values squared (i.e., the "Friston 24-parameter model")[3]. The timeseries extracted from each of the white matter and cerebrospinal fluid were also regressed from the data. Seed-based functional connectivity was then undertaken. The seeds were selected based on brain regions showing a between-group structural difference[4], and created based on the peak coordinates and 4mm radius spherical ROI. The seed-to-voxel correlation maps, constrained to the DMN/ECN mask described above, were transformed to Fisher z-maps for group comparison. Between-group inference was undertaken using a cluster-level FWE correction at $p<0.05$ threshold.

The grey matter volumes of brain areas which showed group differences were extracted (with 4mm radius sphere ROI) and correlated with MASK scores in SDc individuals. Similarly, the connectivity coefficients were extracted for regions showing group-differences in functional

connectivity with the selected seeds, and correlation analyses were conducted between the connectivity strength and MASK scores in SDc and IDED measures in all participants. Pearson correlation if data was normally distributed and Spearman correlation if data was not normally distributed.

Supplementary references

1. Shirer, W. R., Ryali, S., Rykhlevskaia, E., Menon, V. & Greicius, M. D. Decoding subject-driven cognitive states with whole-brain connectivity patterns. *Cereb. Cortex* **22**, 158-165, doi:10.1093/cercor/bhr099 (2012).
2. Yan, C.-G., Wang, X.-D., Zuo, X.-N. & Zang, Y.-F. DPABI: data processing & analysis for (resting-state) brain imaging. *Neuroinformatics* **14**, 339-351, doi:10.1007/s12021-016-9299-4 (2016).
3. Friston, K. J., Williams, S., Howard, R., Frackowiak, R. S. J. & Turner, R. Movement-related effects in fMRI time-series. *Magn. Reson. Med.* **35**, 346-355, doi:10.1002/mrm.1910350312 (1996).
4. Wang, J. *et al.* Increased gray matter volume and resting-state functional connectivity in somatosensory cortex and their relationship with autistic symptoms in young boys with autism spectrum disorder. *Frontiers in Physiology* **8**, 588, doi:10.3389/fphys.2017.00588 (2017).

Supplementary Tables

Supplementary Table 1. Demographic, clinical and cognitive measures of participants

	SDc (N=12)		TDC (N=9)				
	Mean	*SD*	*Mean*	*SD*	*t/Chi-squared*	*p*	Cohen's *d*
Age	12.15	2.67	13.49	1.85	1.29	0.211	-0.58
Gender (male: female)	7:5		6:3		0.15	0.697	
MASK total	153.50	12.00	71.33	8.34	17.55	<0.001	7.95
MASK social/pragmatic symptoms	82.17	11.05	38.22	6.06	10.74	<0.001	4.93
MASK positive schizotypal symptoms	71.33	10.68	33.11	3.66	10.24	<0.001	4.79
IDED Stages completed	7.89	1.05	9.00	0.00	3.16	0.006	-1.50
IDED intra-dimensional errors	2.00	1.12	1.67	0.87	0.71	0.490	0.33
IDED extra-dimensional errors	18.22	12.44	6.22	6.36	2.58	0.020	1.21
IDED extra-dimensional reversal errors	3.00	5.55	4.89	7.01	0.63	0.535	-0.30

SDc, children with schizotypal disorder; TDC, typically-developing children; MASK, Melbourne Assessment of Schizotypy in Kids; IDED, intra-/extra-dimensional set-shifting. For IDED task, there were 9 participants' data in each group.

Supplementary Table 2. Group comparison on brain structure and resting-state functional connectivity

Brain areas	Hemisphere	Peak Coordinates (x y z)	T	Cluster size (voxels)	Cohen's *d*
**Brain structure**					
SDc > TDC					
None					
TDC > SDc					
Superior Frontal Gyrus (BA8)	Left	-12 45 42	7.06	131	3.24
Superior Frontal Gyrus (BA8)	Right	27 32 42	5.40	130	2.48
Medial Frontal Gyrus (BA10)	Left	-8 62 9	5.32	124	2.44
**Resting-state functional connectivity**					
SDc > TDC					
Seed: Superior Frontal Gyrus [-12 45 42]					
Inferior parietal lobe (BA39)	Right	45 -66 45	6.01	74	2.76
TDC > SDc					
None					

SDc, children with schizotypal disorder; TDC, typically-developing children.

Supplementary figures

Supplementary Fig. 1 Structural difference between SDc and TDC with large effect size. These maps shows brain strucuture difference between SDc and TDC with large effect size (Cohen's d > 0.8), pointing to potentially more extensive DMN and ECN involvement beyond the power of this pilot work to infer definitively. SDc, children with schizotypal disorder; TDC, typically developing children; DMN, default mode network; ECN, executive central network.

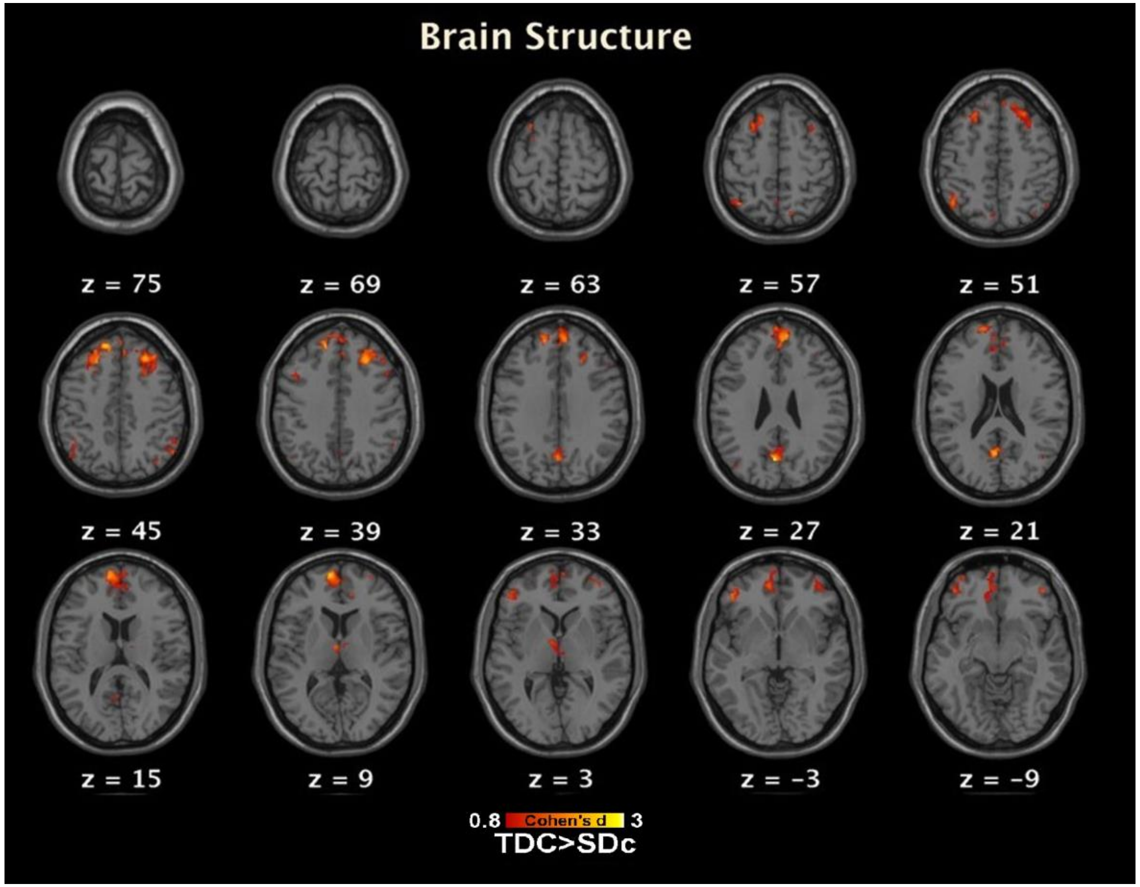

Supplementary Fig. 2 Functional connectivity difference between SDc and TDC with large effect size. These maps shows resting state functional connectivity difference between SDc and TDC with large effect size (Cohen's d > 0.8), the seed is the superior frontal gyrus (centered at [-12,45,42]), the functional connectivity is within DMN and ECN. These maps provide preliminary indications of widespread abnormalities in DMN-ECN interactions in SDc. SDc, children with schizotypal disorder; TDC, typically developing children; DMN, default mode network; ECN, executive central network.

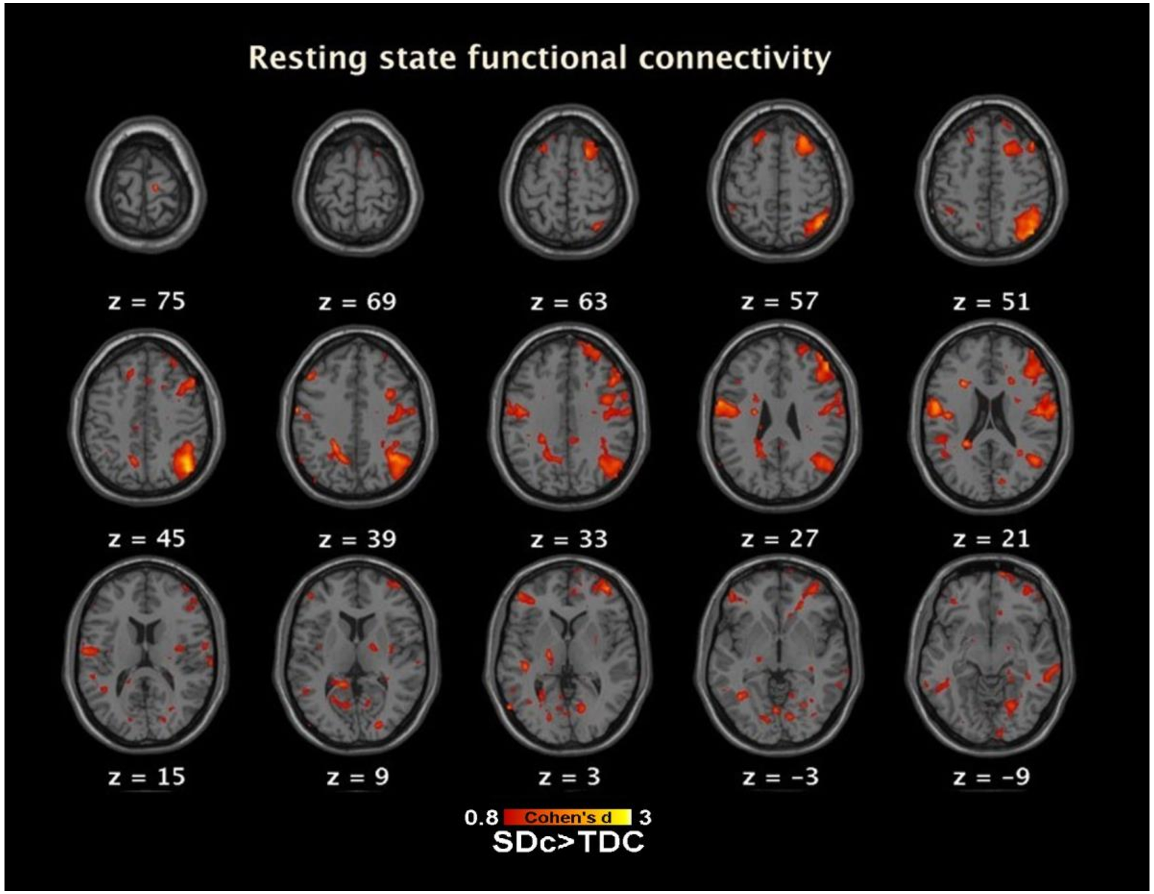

Supplement: Supplementary file 1 — Supplementary_materials [file 41537_2020_95_MOESM1_ESM.pdf]
